# Supplementary material for: Plastic and Placenta: Identification of Polyethylene Glycol (PEG) Compounds in the Human Placenta by HPLC-MS/MS System
Source: Int J Mol Sci. 2022 Oct 22;23(21):12743. doi: 10.3390/ijms232112743 (PMC9656682; doi:10.3390/ijms232112743)
Supplement: Supplementary file 1 [file ijms-23-12743-s001.zip › ijms-1914723-supplementary.pdf]

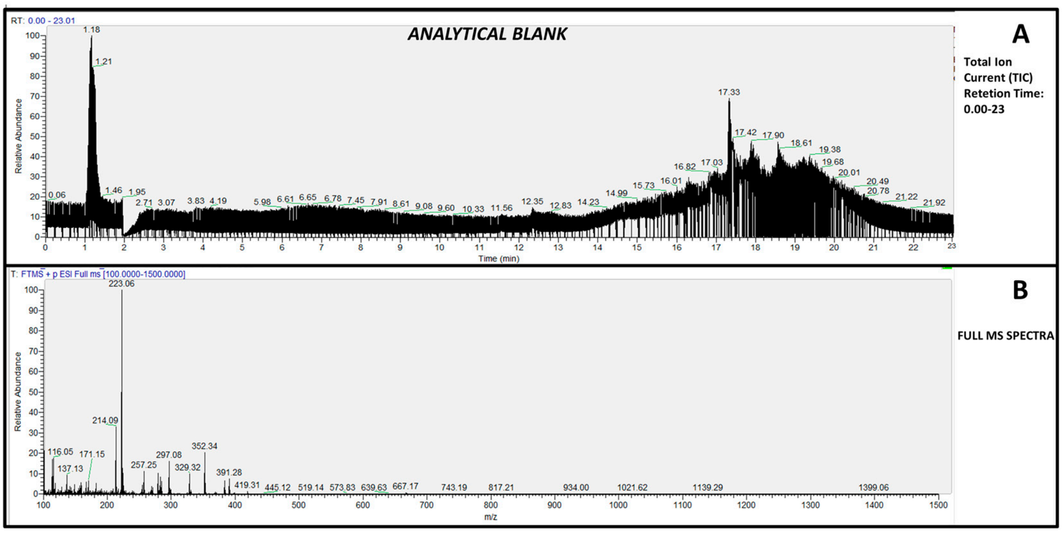

Figure S1. **A** represents the TIC of the analytical blank represented by a mixture of water and 5% formic acid inside of glass vials (the same buffer used for the analysis all the samples). **B** represents the spectrum in ms/ms at RT 9.2 min of the analytical blank. The analytical blanks were analyzed, before carrying out the analyzes of the 36 samples and between one sample and the next.

Table S1. Identification of PEG polymers, 4 to 10 ethylene oxide monomers (EO-4 to EO-10) by HPLCS-MS / MS

MEASURED MASS	PUTATIVE FORMULA	PUTATIVE IDENTIFICATION	CALCULATED EXACT MASS
195.1228	$C_8H_{18}O_5$	PEG-EO4	195.1227
239.1489	$C_{10}H_{22}O_6$	PEG-EO5	239.1489
283.1752	$C_{12}H_{26}O_7$	PEG-EO6	283.1751
327.2018	$C_{14}H_{30}O_8$	PEG-EO7	327.2013
371.2284	$C_{16}H_{34}O_9$	PEG-EO8	371.2276
415.2542	$C_{18}H_{38}O_{10}$	PEG-EO9	415.2538
459.2801	$C_{20}H_{42}O_{11}$	PEG-EO10	459.2800
